# Histone modifications in drug-resistant cancers: From a cancer stem cell and immune evasion perspective

**DOI:** 10.1038/s12276-023-01014-z

**Published:** 2023-07-03

**Authors:** Ming Li Jin, Kwang Won Jeong

**Affiliations:** Gachon Research Institute of Pharmaceutical Sciences, College of Pharmacy, Gachon University, 191 Hambakmoero, Yeonsu-gu, Incheon, 21936 Republic of Korea

**Keywords:** Cancer stem cells, Cancer microenvironment

## Abstract

The development and immune evasion of cancer stem cells (CSCs) limit the efficacy of currently available anticancer therapies. Recent studies have shown that epigenetic reprogramming regulates the expression of characteristic marker proteins and tumor plasticity associated with cancer cell survival and metastasis in CSCs. CSCs also possess unique mechanisms to evade external attacks by immune cells. Hence, the development of new strategies to restore dysregulated histone modifications to overcome cancer resistance to chemotherapy and immunotherapy has recently attracted attention. Restoring abnormal histone modifications can be an effective anticancer strategy to increase the therapeutic effect of conventional chemotherapeutic and immunotherapeutic drugs by weakening CSCs or by rendering them in a naïve state with increased sensitivity to immune responses. In this review, we summarize recent findings regarding the role of histone modifiers in the development of drug-resistant cancer cells from the perspectives of CSCs and immune evasion. In addition, we discuss attempts to combine currently available histone modification inhibitors with conventional chemotherapy or immunotherapy.

## Cancer drug resistance: Preventing modification of chromatin proteins

Understanding how modification of histones, structural proteins that support chromatin and affect gene expression, helps cancer stem cells interact with immune cells will help fight drug-resistant cancers. An important mechanism of cancer treatment resistance is histone modification. Ming Li Jin and Kwang Won Jeong at Gachon University in Incheon, South Korea, reviewed recent research into histone modifications in drug-resistant cancers. Much is known about how histone-modifying enzymes remodel the tumor micro-environment, allowing cancer stem cells to proliferate and block immune cell activity. Drugs that inhibit histone-modifying enzymes show great promise, but it is difficult to identify the precise mechanisms involved. Research should also address why inhibitors are less effective in some cancers, and how a patient’s unique tumor micro-environment could be analyzed to provide personalized precision medicine.

## Introduction

Acquired resistance to anticancer drugs is commonly observed in most cancer types and is caused by both genetic and epigenetic intratumoral heterogeneity. Intratumoral heterogeneity refers to the formation of genetically diverse cell clusters, inducing the development of clones of drug-resistant cancer cells, including cancer stem cells (CSCs), after drug treatment. The development of anticancer drug resistance is also facilitated by heterogeneous complex and dynamic interactions in the tumor microenvironment (TME)^1^.

Previous studies have reported that epigenetic reprogramming regulates the expression of characteristic marker proteins and tumor plasticity associated with cancer cell survival and metastasis in CSCs^2^. Notably, CSCs have additional unique mechanisms to evade external immune cell attacks. The mechanisms used by the immune checkpoint (IC) and IC ligands to mediate immunosuppression and facilitate CSC stemness are well established. Moreover, an increased understanding of tumor-induced immune tolerance has led to innovative anticancer therapeutic strategies based on immune stimulation, such as cancer immunotherapy, including key drugs such as immune checkpoint inhibitors (ICIs) that positively regulate immune cell activation. ICIs have yielded promising results in melanoma treatment with subsequently positive effects in other cancer types^3^.

Self-renewal and tumor heterogeneity within subpopulations enables CSC survival against chemotherapy, leading to metastasis and tumor recurrence^4^. Immunotherapy has also yielded diverse results due to variable patient responses. Some patients treated with ICIs displayed a better prognosis due to tumor-specific immunological memory induction; however, a significant number of patients demonstrated ICI resistance, with recurrence even seen in patients with earlier responses^5^. Several theories have been postulated to explain the chemoresistance of CSCs and the unresponsiveness of ICIs. Epigenetic changes in tumor and immune cells are one of the key mechanisms proposed thus far^6,7^. DNA methylation and histone modifications can cause CSCs to increase stemness, reduce the expression of essential tumor-associated antigens on cancer cells recognized by the immune system, and modify IC expression^8^. Additionally, alterations in chemokine expression induced by histone-modifying enzymes lead to an immunosuppressive TME that contributes to the immune escape of CSCs^9^. Thus, targeting epigenetic regulators associated with the survival and immune evasion of CSCs is a novel therapeutic approach to effectively prevent cancer and has become a prominent field of interest, with a special focus on the regulation of histone modification, which could diversify therapeutic strategies for drug-resistant cancer cells. In this review, we summarize the latest findings on the roles of histone modifications in acquired drug resistance and immune evasion in CSCs and provide recent clinical treatment strategies for resistant cancer.

## Aberrant signaling in CSCs regulated by histone modifications

**Fig. 1:**
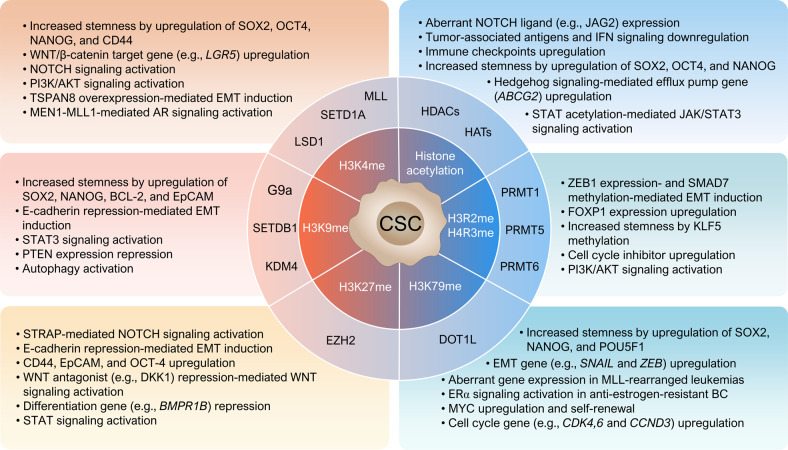
Aberrant histone modifications in cancer stem cells affecting drug resistance. Cancer stem cells exhibit resistance to chemotherapy and radiation therapy through various intrinsic factors, including survival-promoting and anti-apoptotic signals and overexpression of drug efflux pumps. During resistance development, histone modification regulators create favorable conditions for CSC survival, self-renewal, and EMT regulation by modifying various signal transduction pathways, including the WNT, NOTCH, JAK/STAT, hedgehog, and PI3K/AKT/mTOR pathways in CSCs. Histone H3K4 methylation by SETD1A promotes the aberrant expression of stemness genes (e.g., SOX2 and OCT4). Epigenetic silencing of the CHD1 and PTEN genes by histone H3K9 and K27 methylation promotes EMT and induces the development of resistance against EGFR tyrosine kinase inhibitors. HDACs are involved in CSC stemness maintenance and the regulation of NOTCH, STAT3, and AKT signaling and are thus considered representative epigenetic target molecules for the treatment of cancers refractory to existing anticancer therapies.

Compared with progenitor cells, most CSCs exhibit increased drug resistance against chemotherapy and radiotherapy due to various factors, such as survival-promoting and anti-apoptotic signaling, overexpressed drug efflux pumps, intracellular drug-inactivating enzymes, enhanced DNA repair, and tumor niche^2^. Thus, a deeper understanding of CSCs leads to better implementation of existing anticancer therapies and the identification of new approaches to specifically target CSCs. In addition, CSCs modulate histone modifications to induce favorable conditions for cell survival and self-renewal through aberrant modification of various signaling pathways in normal cells, including the WNT, NOTCH, Janus kinase (JAK)/signal transducer and activator of transcription (STAT), hedgehog, and phosphatidylinositol-3-kinase (PI3K)/AKT/mammalian target of rapamycin (mTOR) signaling pathways (Fig. 1).

The WNT signaling pathway is one of the most evolutionarily conserved signaling pathways, and its activation by WNT ligands plays critical roles in regulating stem cell differentiation and pluripotency^10^. Histone modification is a major mechanism underlying the dysregulated WNT/β-catenin pathway in cancer. For example, in glioblastoma, the decreased acetylation of histone H4K16 and increased trimethylation of histone H3K27 in the *DKK1* gene promoter, a WNT antagonist, suppresses its expression, resulting in WNT signaling overactivation, which assists in CSC maintenance and tumorigenesis. Moreover, the recruitment of achaete-scute family bHLH transcription factor 1 (ASCL1) at H3K4me1 in the *DKK1* gene enhancer region promotes repressive chromatin configuration in the form of a poised enhancer^11,12^. Thus, histone modifications alter the WNT signaling pathway.

Another mechanism of acquired chemoresistance in CSCs is via NOTCH signaling activation that induces self-renewal, epithelial-to-mesenchymal transition (EMT), and overexpression of efflux pumps such as ATP binding cassette subfamily C member 1 (ABCC1/MRP1)^13,14^. Jin et al. described the mechanism underlying the epigenetic activation of the NOTCH pathway by the serine-threonine kinase receptor-associated protein (STRAP) in CSCs to promote their proliferation and maintenance. STRAP reduces the histone methylation of H3K27 at the *HES1* and *HES5* promoter sites by inhibiting the association between the enhancer of zeste 2 polycomb repressive complex 2 subunit (EZH2) and SUZ12 subunits of the polycomb repressive complex (PRC)2, ultimately activating gene expression^15^. Moreover, abnormal overexpression of NOTCH ligands, including JAG2, due to histone acetylation at their promoter sites has been observed in multiple myeloma cells^16^.

The hedgehog (Hh) signaling pathway is activated by ligands including desert Hh, sonic Hh, and Indian Hh, and their association with Patched (PTCH) leads to the activation and nuclear translocation of downstream transcription factors, which induces the expression of genes involved in CSC survival, proliferation, and drug resistance^17^. The GLI1 and GLI2 proteins are regulatory transcription factors of Hh signaling and require deacetylation by HDAC1 to activate transcription^18^. Hh signaling directly regulates the efflux pump *ABCG2* gene, where epigenetic histone modifications such as H3 acetylation, H3K4 trimethylation, and H3S10 phosphorylation upregulate its expression^19^. Additionally, in malignant rhabdoid tumors, the inactivation of SNF5, which directly interacts with GLI1 to suppress the expression of Hh target genes, including *GLI1* and *cyclin D*, contributes to the activation of Hh signaling.

The JAK-STAT signaling pathway is the main axis of cytokine-mediated immune responses and is frequently dysregulated in CSCs, contributing to their stemness and self-renewal properties. Activation of the JAK/STAT3 signaling pathway also increases tumorigenesis and metastasis via enhanced EMT and CSC transition, leading to cancer chemoresistance^20^. The histone acetyltransferase p300 (KAT3B) plays a role in cytokine-induced STAT3 activation by acetylating it at Lys685^21^.

Aberrations in the PI3K/AKT/mTOR pathway have been shown to induce stemness, EMT, autophagy, and acquired resistance in CSCs^22^. Changes in the chromatin landscape caused by histone modifiers exert direct or indirect effects on CSC survival, and some of these modifiers are regulated by the PI3K/AKT signaling pathway. For example, AKT-mediated phosphorylation at Ser21 of EZH2 inhibits its activity. AKT also phosphorylates p300 and CREB-binding protein (CBP/KAT3A) to stimulate HAT activities, allowing them to participate in other signaling pathways in CSCs^23^.

E-cadherin, encoded by the *CDH1* gene, regulates the TME as an adherent junction protein that maintains cells in an epithelial state, and its downregulation induces EMT^24^. E-cadherin expression is regulated by SNAIL, TWIST, and zinc finger E-box binding homeobox 1 (ZEB1/ZEB2) transcription factors^25^. The EZH2 and PRC2 complex binds to SNAI1 for recruitment to the *CDH1* promoter, and methylation of H3K27 suppresses E-cadherin expression^26^. KDM7B (PHF8) induces EMT by enhancing SNAIL1 and ZEB1 expression by removing the repressive histone marks H3K9me1/2, H3K27me2, and H4K20me19^27^. Another mechanism of EMT regulation via histone modification is by LSD1 (KDM1A), which is overexpressed in various cancer types. By removing the mono- and dimethyl groups from histones K4 and K9, LSD1 regulates chromatin configuration and gene expression^28^. LSD1-mediated AKT activation promotes EMT in a CRC cell line with a PIK3CA mutation^29^, and another study using a different CRC cell line showed that LSD1 reduced the level of H3K9me2 in the promoter region of the EMT gene *TSPAN8*, leading to its overexpression^30^.

## CSC-immune cell crosstalk in immune evasion

CSCs evade T-cell-mediated antitumor immune surveillance through various mechanisms, including suppression of T-cell activation, altered expression of major histocompatibility complex (MHC)-I at the transcriptional and posttranscriptional levels, downregulation of tumor-associated antigens (TAAs), and overexpression of ICs such as programmed cell death 1 ligand 1 (PD-L1) and Galectin-9 (Gal-9)^8^. MHC-I downregulation affects CD8^+^ T-cell activation that targets TAAs expressed by CSCs. WNT/β-catenin signaling acts on STT3, which is responsible for the glycosylation and stabilization of PD-L1, resulting in increased PD-L1 levels in CSCs, thereby contributing to CSC evasion of T-cell immune surveillance^31^. In hypoxic TME conditions, the expression of PD-L1 and vascular endothelial growth factor (VEGF) in CSCs is upregulated, and the expression of the T-cell inhibitory receptor TIM-3 is promoted via VEGF^32^. The overexpression of immune checkpoints such as PD-L1 and Gal-9 results in their association with corresponding receptors (e.g., PD-1 and TIM-3) in T cells to suppress T-cell proliferation and cytokine production and induce T-cell exhaustion, leading to CSC evasion of cytotoxic T cells^8^.

Mature dendritic cells (DCs) present TAAs and express costimulatory molecules that activate T-cell-mediated immune responses^33^. CSCs inhibit DC antitumor responses through various mechanisms, including transforming growth factor (TGF)-β release. Specifically, CSCs prevent the recruitment of CD103^+^ DCs to tumors^34^ and inhibit their maturation while promoting differentiation into immunosuppressive regulatory DCs (DCreg)^35^ and initiating the development of PD-1^+^ DCs that inactivate CD8^+^ T cells^36^. As an upregulated IC in chronic lymphocytic leukemia, breast cancer (BC), non-small-cell lung cancer (NSCLC), and CRC, CD200 induces immunological tolerance by negatively regulating DCs, macrophages, and T cells that express its receptors^37^.

The crosstalk between tumor-associated macrophages (TAMs) and CSCs supports the survival of CSCs and the formation of an immunosuppressive TME^38^. The niche of CSCs involves a unique TME with various cells, including fibroblasts and endothelial and immune cells, and is enriched in C-C motif chemokine 2/5 (CCL2/5), interleukins (ILs), periostin (osteoblast-specific factor, OSF-2), TGF-β, and colony-stimulating factor 1 (CSF1)^39^, which induce the conversion of protumorigenic macrophages into the immunosuppressive M2 or TAM phenotype^40^. TAM-released factors, including WNT, TGF-β, and VEGF, induce cancer stemness, immunosuppressive TME, EMT, and cancer metastasis^41^. TAMs also stimulate the expression of PD-L1 in CSCs and PD-1 in T cells to reduce T-cell-mediated cytotoxicity^42^.

Myeloid-derived suppressor cells (MDSCs) secrete cytokines and chemokines in the TME to assist in the formation of an immunosuppressive niche and reduce the efficacy of immunotherapy^43^. The mTOR signaling pathway is used by CSCs to promote the release of granulocyte-macrophage colony-stimulating factors (GM-CSFs) to induce tumor infiltration of MDSCs^44^. MDSCs in the TME release IL-6 and nitric oxide to induce the expression of CSC markers such as NANOG, OCT4, and SOX2 through epigenetic regulation^45^ while promoting the activation of regulatory T cells (Tregs) through the release of TGF-β^46^.

As an immunosuppressive T-cell-derived subpopulation, Tregs promote CSC immune evasion by interacting with them in the immunosuppressive TME to inhibit the effects of cancer immunotherapy. TGF-β produced by CSCs mediates tumor infiltration by Tregs, which is associated with a poor survival rate^47^. In addition, CSCs increase CCL1 expression to recruit Tregs to the TME via epigenetic mechanisms that reduce the level of H3K27me3 at the *CCL1* promoter site^48^. CSCs also evade T-cell-induced apoptosis by differentiating uncommitted CD4^+^ T cells into Tregs^49^. In particular, Tregs in the hypoxic TME release VEGF to induce CSC stemness and angiogenesis, ultimately promoting EMT^50,51^.

Natural killer (NK) cells express killer cell lectin-like receptor K1 (NKG2D), Fas ligand (FASL), and TNF-related apoptosis-inducing ligand (TRAIL), which are activated by their respective binding factors to selectively kill MHC-I-negative CSCs^52^. NK cell-mediated cell lysis has been reported in MHC-I-negative colon CSCs (or cancer-initiating cells) that express NKG2DL^53^ and in MHC-1-negative ovarian CSCs that express ligands for the activating receptors NKp30 and NKp44^54^. However, CSCs of some ovarian and renal carcinoma patients display upregulated MHC-I molecules, making them less susceptible to NK cell-mediated cell lysis^55,56^. Interestingly, latent competent cancer cells expressing SOX2/SOX9 increase dormant CSCs (or latency-competent cancer cells) that downregulate NKG2DL through a unique mechanism that produces the WNT inhibitor DKK1, thus evading NK cell-mediated immunity^57^.

**Fig. 2:**
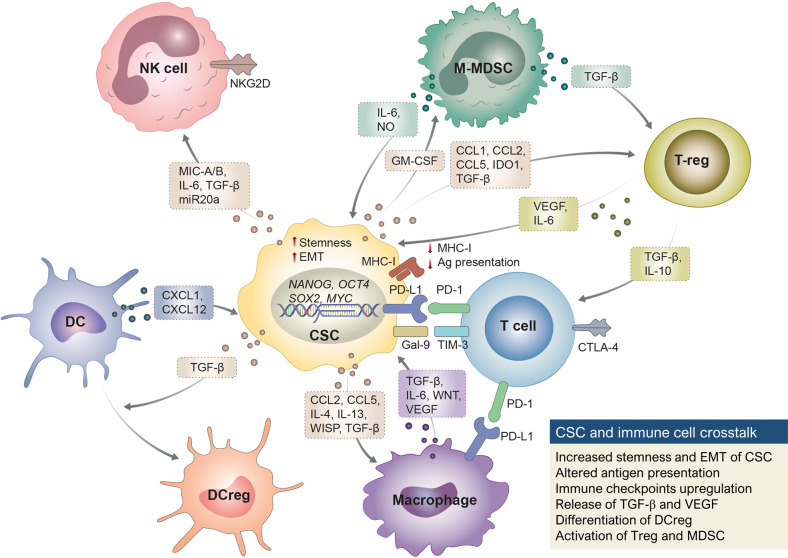
Immune evasion by crosstalk between immune cells and CSCs in the tumor microenvironment. CSCs inhibit T-cell immune-mediated cytotoxicity by downregulating MHC-I and TAA expression and overexpressing immune checkpoints, such as PD-L1. The overexpression of immune checkpoints results in their association with corresponding receptors (e.g., PD-1 and TIM-3) in T cells to suppress T-cell proliferation and cytokine production and induce T-cell exhaustion, leading to CSC evasion of cytotoxic T cells. CSC-derived TGF-β/CCL interferes with the recruitment of DCs to the tumor site, promotes differentiation into DCregs, and induces the differentiation of macrophages from M1 to M2. VEGF, WNT, and TGF-β secreted from tumor-associated macrophages promote the stemness and EMT of CSCs. MDSCs secrete IL-6 and NO to induce the expression of CSC markers (e.g., NANOG, OCT4, and SOX2) and TGF-β to promote the activation of regulatory T cells (Tregs). Treg-derived VEGF induces the survival and EMT of CSCs. CSC-derived IL-6 and TGF-β downregulate NK-activating receptors, and CSCs inhibit NK cell activation through the release of MIC A/B and CD155 inhibitory activating receptors.

Interferon (IFN) is a potent antitumor cytokine that prevents CSC expansion and suppresses tumor-initiating properties. In addition, IFN-stimulated genes suppress the chemoresistance of CSCs^58^. However, CSCs develop mechanisms to resist the antitumor effect of IFN and induce the expression of CSC markers and immune evasion using IFN signaling^59,60^. This dual role of IFNs may depend in part on the duration and concentration of IFN exposure^61,62^. Suboptimal type-I IFN signaling induced by immunogenic cell death (ICD) did not lead to therapeutic anticancer immunity but rather promoted tumor progression through the expansion of the CSC population with characteristic immune evasion^63^. Similarly, while a high concentration of IFN-γ induced apoptosis in NSCLC through the JAK1/STAT1/caspase pathway, a low concentration of IFN-γ enhanced the properties of CSCs through the ICAM1/PI3K/AKT/NOTCH1 pathway^64^. As described above, the crosstalk between various immune cells and CSCs contributes to the formation of an immunosuppressive TME and immune evasion of CSCs, ultimately leading to adaptive resistance against cancer immunotherapy (Fig. 2).

## Histone modifications in CSC drug resistance

### Histone lysine methylation

The functions of SET domain family proteins in drug-resistant or refractory cancers are well documented^65^. The mixed-lineage leukemia gene (*MLL1/KMT2A*) fused by chromosomal translocation is a prominent cause of aggressive leukemia in both children and adults^66^. Mechanistically, the MLL1 fusion protein in acute myeloid leukemia (AML) causes abnormal recruitment of histone modification enzymes, including DOT1L (KMT4) and protein arginine methyltransferase (PRMT) 1, at target genes for epigenetic reprogramming, conferring stem cell-like properties^67,68^. Recently, developed MLL inhibitors prevent the interaction between MEN1 and MLL1 to downregulate the expression of their target genes, thereby exerting a therapeutic effect on leukemia^69^. The MEN1-MLL1 association also regulates androgen receptor signaling in castration-resistant prostate cancer (CRPC). MI-136, a selective MEN1-MLL1 inhibitor, blocks this signaling to suppress tumor growth^70^. In addition, MLL1 regulates the expression of *LGR5*, a target gene of WNT/β-catenin and a stem cell gene in intestinal stem cells. LGR5^+^ intestinal stem cells and human colon carcinoma with elevated MLL1 and β-catenin expression levels are associated with poor survival^71^. In contrast, MLL3 (KMT2C), which is frequently lost in myelodysplastic syndrome (MDS), AML, aggressive nasopharyngeal carcinoma, and CRC, acts as a myeloma inhibitor that suppresses the self-renewal of hematopoietic stem cells and engages in differentiation via IL-1 stimulation^72^. MLL4 (KMT2D) is the gene that most frequently exhibits mutations in oral squamous cell carcinoma, and MLL4 knockdown decreases the expression of CD133 and β-catenin, colony formation, metastasis, and invasion to delay tumor growth^73^. In hematopoietic tissues, MLL5 (KMT2E) is involved in terminal myeloid differentiation and hematopoietic stem cell self-renewal^74^.

Recently, we reported the critical role of SETD1A (KMT2F) in CSC stemness in tamoxifen-resistant BC and CRPC. In tamoxifen-resistant BC, SETD1A expression is upregulated, which directly regulates the expression of SOX2, which plays a crucial role in the acquisition of stemness and resistance to tamoxifen^75^. Similarly, SETD1A expression is higher in metastatic CRPC (mCRPC) cells than in primary prostate cancer cells, whereas the expression of the stem cell factor OCT4 and transcription factor FOXM1 is upregulated^76^. Additionally, SETD1A allows NSCLC cells to maintain the characteristics of CSCs through WNT/β-catenin signaling and to develop resistance against anticancer drugs such as cisplatin^77^.

SETDB1 (KMT1E) is overexpressed in melanoma, BC, and lung cancer, is involved in stem cell maintenance and is considered an effective target for cancer therapy. Zhang et al. showed that SETDB1 is closely associated with EMT in BC stem cells, as it directly regulates STAT3 signaling^78^. In addition, the blockade of SETDB1 significantly increased the sensitivity of KRAS-mutant CRC to cetuximab treatment, demonstrating that it could be a potential anticancer target^79^. However, the lack of selective inhibitors remains an additional obstacle for studies on SETDB1.

G9a (EHMT2/KMT1C), which is responsible for mono- and dimethylation of H3K9, is a representative histone methylation enzyme that induces the epigenetic silencing of tumor suppressor genes, including *TP53*, *CDH1, DUSP5*, and *PTEN*, contributing to cancer metastasis and the maintenance of the malignant phenotype^80^. G9a interacts with SNAI1 at the *CDH1* promoter and promotes EMT and lymph node metastasis by suppressing the expression of E-cadherin via H3K9 methylation^81^. In addition, G9a in lung cancer increases H3K9me2 levels at the *PTEN* promoter to suppress its transcription, thereby activating the AKT signaling pathway and contributing to the development of resistance against EGFR tyrosine kinase inhibitors (EGFR-TKIs). These results confirmed that the G9a inhibitor UNC0638 suppressed the growth of drug-resistant cancer cells^82^. In glioma stem-like cells, which are generally resistant to conventional therapy, autophagy plays a critical role in stemness, and G9a acts on the promoters of genes associated with autophagy (*MAP1LC3B* and *WIPI1*) and differentiation (*GFAP* and *TUBB3*). The G9a-specific inhibitor BIX-01294 upregulated the genes associated with autophagy and differentiation in glioblastoma CSCs to induce autophagy-dependent differentiation of glioma stem-like cells^83^.

Tazemetostat, which was recently approved as an inhibitor of EZH2, a histone H3K27 selective methyltransferase, has shown positive effects not only in epithelioid sarcoma but also in a variety of drug-resistant cancers. In SMARCB1 (*a.k.a*. SNF5)-deleted malignant rhabdoid tumors, tazemetostat induces the expression of genes related to neuronal differentiation and cell cycle inhibition while suppressing the Hh pathway genes. Notably, malignant rhabdoid tumors in animal models displayed dose-dependent degeneration after treatment with tazemetostat, and tumor regrowth was prevented even after the discontinuation of administration^84^. In papillary thyroid cancer, abnormal H3K27 trimethylation plays an important role in BRAF^V600E^-MAPK-induced differentiation and drug resistance. Combination treatment with tazemetostat and a MAPK inhibitor resulted in a reduction in global H3K27 trimethylation, leading to the differentiation of papillary thyroid cancer with BRAF^V600E^ ^85^. Resistance to receptor tyrosine kinase inhibitors (RTKis) is a major obstacle in the treatment of clear cell renal cell carcinoma. Overexpression of EZH2 promotes global kinase phosphorylation, which induces acquired and intrinsic resistance. Treatment with the EZH2 inhibitor EPZ011989 reduced kinase phosphorylation and activated tumor suppressors to reverse resistance to sunitinib^86^.

DOT1L, a histone H3K79 methyltransferase, plays an important role in the proliferation, self-renewal, and metastasis of BC cells resistant to antihormone therapy^87^. In anti-estrogen-resistant BC, blocking DOT1L affects ERα-dependent transcription, including silencing of *ESR1* and *FOXA1* genes, with a consequent inhibitory effect on tumor growth both in vitro and in vivo^88^. A recent study demonstrated that DOT1L is the main regulator of CSCs, with roles in the expression of MYC in ALDH1^+^ triple-negative BC (TNBC) and the maintenance of self-renewal. The DOT1L inhibitor EPZ-5676 reduced tumorsphere formation and ALDH1^+^ cells in vitro and inhibited tumor-initiating stem cells and metastasis of ALDH1^+^-derived tumor xenografts in vivo^89^.

### Histone arginine methylation

PRMT1, a type I protein arginine methyltransferase, catalyzes the production of asymmetric dimethylarginine and plays a role in various cellular processes, including the generation of hematopoietic and tumor cells^90^. PRMT1 promotes EMT and CSC properties by activating *ZEB1* gene transcription via the asymmetric dimethylation of H4 (H4R3me2as) at the *ZEB1* promoter in BC cells^91^. Additionally, PRMT1 acts as a crucial mediator of TGF-β signaling and promotes TGF-β-induced EMT and the generation of epithelial stem cells through SMAD7 methylation^92^.

PRMT5 catalyzes the production of symmetric dimethyl arginine and is involved in various cellular processes, including pluripotency and tumorigenesis. In patient-derived BC stem cells, PRMT5 generates H3R2me2s at the *FOXP1* promoter, which is recognized by WDR5, a component of the SET1 complex that consequently promotes gene expression^93^. Treatment of liver CSCs with a PRMT5 inhibitor (DW14800) results in the inhibition of *HNF4A* gene expression by reducing H4R3me2, resulting in the reconstruction of hepatocyte-specific properties and antitumor effects^94^. In addition, PRMT5 in basal-like BC cells promotes the maintenance and proliferation of BC stem cells through arginine methylation of KLF5^95^. Recently, PRMT5 has emerged as a promising target for glioblastoma treatment. The critical influence of PRMT5 on CSC growth in BC and glioblastoma has been verified using a PRMT5 inhibitor (e.g., GSK3203591 or LLY-283)^93,96^.

Inhibition of PRMT6 in cancer cells upregulates tumor suppressor genes. The depletion of PRMT6 results in increased p21, p27, and CD44 and downregulated MMP-9 expression and PI3K/AKT/mTOR signaling in CRPC, thus inducing sensitivity to chemotherapy, and the methylation of p21 by PRMT6 (R156) reduces chemosensitivity in CRC cells^97,98^. Therefore, this strategy can be used to improve the efficacy of conventional therapies for drug-resistant cancers. In addition, PRMT6 assists in the maintenance of CSC characteristics in glioblastoma through methylation of the regulator of chromosome condensation 1 (RCC1) protein. Hence, EPZ020411, a PRMT6-specific inhibitor, inhibits the methylation of the arginine residue of RCC1 to maximize the positive effects of radiotherapy in a glioblastoma xenograft model^99^.

### Histone demethylation

LSD1 interacts with OCT4 in CSCs to turn the enhancer site of the pluripotency gene (PpG) into a ‘primed’ state with sensitivity to reactivation, and abnormally increased PpG expression leads to the increased tumorigenicity of CSCs^100^. Liu et al.^101^ showed that the increased LSD1 expression in CSCs of hepatocellular carcinoma is critical for maintaining self-renewal and tumorigenicity through activated NOTCH signaling and that LSD1 overexpression in non-CSCs is sufficient to induce self-renewal. In contrast, inactivation of LSD1 leads to the downregulation of SOX2 and OCT4 and the induction of differentiation genes such as *BMP2*^102^. In BC cells, LSD1 regulates BC stem cells by modulating self-renewal, EMT, and anticancer drug resistance^103^. Hence, combined treatment with an LSD1 inhibitor and chemotherapy in an in vivo BC model abolished the mesenchymal signature and promoted an innate M1 macrophage-like immune response^104^. PKC-θ is a determinant of the regulation of epigenetic mesenchymal gene expression in LSD1. Phosphorylation of the serine-111 residue of LSD1 (LSD1-s111p) by protein kinase C-theta (PKC-θ) is critical for LSD1 demethylase activity, and phosphorylated LSD1 is elevated in chemoresistant cancer^111^. The enhancer region of SOX2, which is overexpressed in luminal-B BC cells, is a target site for LSD1. Iadademstat, a known LSD1 inhibitor, suppresses SOX2 expression and mammosphere formation in patient-derived CSCs of multidrug-resistant luminal-B BC^105^. Interestingly, LSD1 in gastric cancer patients can migrate to other gastric cancer cells via small extracellular vesicles^106^. In acceptor cells, the delivered LSD1 not only promoted cancer stemness by inducing the expression of NANOG, OCT4, SOX2, and CD44 but also produced resistance to oxaliplatin. Ongoing research is addressing the prevalence of sEV-derived cancer resistance in other carcinomas, which would be a new mechanism of acquired drug resistance by cancer cells.

In ovarian cancer, KDM3A induces the expression of SOX2, NANOG, and the anti-apoptotic protein Bcl-2 and suppresses the expression of p21^WAF1/CIP1^, a cyclin-dependent kinase inhibitor, thereby regulating stemness and cisplatin resistance^107^. Recently, a KDM4 inhibitor, TACH101, displayed promising results in various tumor models. For instance, the administration of TACH101 reduced the rate of CRC growth and the ratio of cells exhibiting CSC characteristics (CD44^high^ EpCAM^+^), and in different xenograft models of lymphoma, esophageal, gastric, and breast cancers, TACH101 inhibited up to 100% of tumor growth (2022 ASCO Annual Meeting Abstract).

### Histone acetylation

The expanded knowledge of the functions of other HAT family proteins in CSCs has prompted the development of inhibitors for the treatment of drug-resistant cancers. For instance, WM-3835 can chemically inhibit the acetyl-CoA binding site of HBO1 (KAT7 or MYST2), ultimately inhibiting leukemia stem cell growth^108^. CPTH6, an inhibitor of PCAF and GCN5, can reduce CSC markers such as CD133 and ALDH and engage in the autophagy pathway to inhibit the growth of lung cancer stem-like cells^109^. WM-8014 and WM-1119 inhibit KAT6A, an acetyltransferase subunit in the MOZ complex, to suppress proliferation and induce senescence in lymphoma via the p16^INK4a^ and p19^ARF^ pathways^110^. In TNBC, these inhibitors block SMAD3 acetylation, suppress cytokine expression, inhibit stemness, and prevent the recruitment of MDSCs to substantially increase sensitivity to anti-PD-L1 therapy^111^. In addition, the inhibition of KAT6A in ovarian cancer could increase sensitivity to cisplatin, which implies its potential use in combination therapy for the treatment of drug-resistant cancers^112^.

### Histone deacetylation

HDAC1 induces the deacetylation and ubiquitination of SMAD7 required for the maintenance of the epithelial phenotype of CSCs in ovarian cancer, thereby reducing SMAD7 stability^113^. In CSCs in head and neck cancer, HDAC1 controls the acetylated state of GRP78 to increase the CD44^high^/CD24^low^ phenotype and assist in CSC maintenance^114^. Additionally, HDAC1 and HDAC7 increase CSC phenotypes, such as miR-34a, CD44, and CD166, assisting with chemotherapy drug resistance, metastasis, and recurrence in BC and ovarian cancer^115,116^. Indeed, high levels of HDAC1 and HDAC7 have been observed in residual tumor cells with low sensitivity to chemotherapy^117^.

STAT3 signaling mediates the maintenance of CSCs by IL-6 in various cancer types^118^, and deacetylation of the STAT3 Lys685 residue by HDAC3 facilitates the phosphorylation of the Tyr705 residue to activate STAT3^119^. HDAC11, which is upregulated in the stem-like population of NSCLC, interacts with the transcription factor GLI1 to activate the expression of *SOX2*. Moreover, selective HDAC11 inhibitors (FT234 and FT895) efficiently inhibit the growth of drug-resistant lung adenocarcinoma stem cells^120^.

SIRT1, another HDAC, is overexpressed in stem-like cells in CRC to increase the CD133^+^ cell population, expression of stemness-related genes including *TDGF1, NANOG, OCT4, TERT*, and *LIN28*, CSC sphere formation, and tumorigenesis^121^. Inhibition of p53 is involved in this process because SIRT1 can deacetylate the C-terminal Lys120, Lys164, and Lys382 residues of p53 to inhibit its activity^122^. Inhibition of SIRT1 activity has been highlighted as an effective alternative for the removal of imatinib-resistant quiescent leukemia stem cells (BCR-ABL tyrosine kinase inhibitor). Indeed, a SIRT1 inhibitor (tenovin-6) increased the expression of the p53 target gene in chronic myeloid leukemia CD34^+^ cells by activating p53 transcriptional activity^123^.

The HDACs described above are representative epigenetic target molecules for the treatment of cancers refractory to existing anticancer therapies. Accordingly, extensive trials of HDAC inhibitor monotherapy or combination therapy with other drugs for various resistant carcinomas have been conducted, and the role of HDAC inhibitors in reducing CSC aggressiveness has been promising. Early HDAC inhibitors, such as vorinostat and panobinostat, exhibited higher effects than anticipated in a single or combined administration in preclinical models of various drug-resistant cancers, thereby providing a sufficient rationale to initiate clinical trials. Vorinostat reverses cisplatin resistance and reduces the self-renewal ability of CSCs by suppressing NOTCH signaling via miR-34a re-expression in head and neck and pancreatic cancer cells^124,125^. For a more effective application of HDAC inhibitors, a detailed in-depth understanding of their differential mechanisms of action in different cancers refractory to HDAC inhibitors is needed. However, new mechanisms for overcoming resistant cancers have been revealed through various combination therapy attempts (Table 1). Thus, the vast accumulated epigenetic information will provide optimal conditions and combination therapies for the treatment of resistant cancer in the near future, along with the development of immuno-oncology, which will be described later.

**Table 1:** Mechanisms of action of histone modification inhibitors used in single or combination therapy for overcoming drug resistance in cancer.

Histone modifier	Inhibitor	Resistant cancer cell type	Combination therapy	Proposed mechanism of overcoming cancer resistance	Refs.
PRMT5	GSK3203591	Breast cancer stem cell	NA	Downregulation of FOXP1 expression by inhibiting H3R2 methylation at the *FOXP1* promoter	^93^
	DW14800	Liver cancer stem cell	NA	Upregulation of HNF4α expression by reducing H4R3me2s levels	^94^
	LLY-283	Glioblastoma stem cell	NA	Deregulation of alternative splicing, affecting regulators of cell cycle	^96^
PRMT6	EPZ020411	Glioblastoma stem cell	Radiation therapy	Inhibition of arginine methylation of RCC1	^99^
G9a	UNC0638	Neuroblastoma (MYCN-amplified)	EPZ011989	Upregulation of IFN-γ-induced expression of CXCL9 and CXCL10	^131^
	UNC0638	NSCLC (EGFR-TKI resistance)	EGFR-TKI (Erlotinib)	Upregulation of PTEN expression and inhibition of the AKT signaling pathway	^82^
	BIX-01294	Glioma stem-like cell	NA	Upregulation of autophagy and differentiation genes	^83^
EZH2	Tazemetostat	Papillary thyroid cancer (radioiodine-refractory)	Dabrafenib, Selumetinib	Upregulation of iodine-metabolizing gene expression and promotion of radioiodine uptake	^85^
		Malignant rhabdoid tumor (SMARCB1-deleted)	NA	Reductions in global H3K27me3 levels, upregulation of genes related to neuronal differentiation and cell cycle inhibition, and silencing of hedgehog pathway genes	^84^
	GSK503	Melanoma	IL-2cx/anti-CTLA-4 Ab	Downregulation of PD-L1 expression and accumulation of IFN-γ-producing CD8^+^ T cells	^135^
	CPI-1205	Bladder cancer, Melanoma	Ipilimumab	Inhibition of EZH2 expression induced by anti–CTLA-4	^140^
	EPZ011989	Clear cell renal cell carcinoma (sunitinib-resistant)	Sunitinib	Re-expression of tumor suppressors (e.g., DAB2IP and PTPN3) and attenuation of the global kinome reprogramming	^86^
DOT1L	EPZ004777	Breast cancer (anti-estrogen resistant)	NA	Silencing of ERα and FOXA1 and inhibition of ER-dependent signaling	^88^
	EPZ-5676	Triple-negative breast cancer	NA	Downregulation of MYC expression	^89^
LSD1	GSK-LDS1	Melanoma (ICI-refractory)	Anti-PD-(L)1 Ab	Increase in endogenous retroviral elements (ERVs) and activation of the dsRNA-IFN pathway	^144^
	HCI-2509	Triple-negative breast cancer	Anti-PD-1 Ab	Induction of the expression of chemokines (CCL5, CXCL9,10) and PD-L1	^145^
	Iadademstat	Luminal-B breast cancer (multidrug-resistant)		Downregulation of SOX2 expression	^105^
	GSK2879552	HCC stem-like cells (sorafenib-resistant)	Sorafenib	Derepression of WNT antagonist expression	^146^
KDM4D	TACH101	Colorectal cancer stem cell	NA	Decrease in CSC signature (CD44^High^ EpCAM^+^)	Meeting Abstract
KAT6A	WM-1119	Triple-negative breast cancer	Anti-PD-L1 Ab	Inhibition of SMAD3 acetylation, reduction of cytokine expression, inhibition of stemness, inhibition of myeloid-derived suppressor cell (MDSC) recruitment	^111^
KAT7	WM-3835	Leukemia stem cell	NA	Removal of acetyl-CoA binding site of HBO1 (KAT7) and repression of HOXA9,10 expression	^108^
HDACs	Vorinostat	Pancreatic cancer	NA	Inhibition of Notch signaling by upregulating miR-34a expression	^125^
		Relapsed lymphoma (rituximab–chemotherapy-resistant)	NA	Increase in p21 and acetylation of histone H3 leading to G1 cell cycle arrest	^169^
		Small-cell lung cancer (navitoclax-resistant)	Navitoclax	Upregulation of BH3-only pro-apoptotic BCL-2 family protein (e.g., NOXA)	^170^
		Pancreatic cancer	Sorafenib, anti-PD-1 Ab	Reduction of HDAC protein levels, promotion of autophagy, reduction of PD-L1 and IDO-1 levels	^171^
		Squamous cancer cell	5-FU/Cisplatin	Reverted 5FU/cisplatin-induced EGFR nuclear translocation, and induction of cisplatin uptake	^172^
		Lung cancer (EGFR-C797S-mutated)	Brigatniib	Reduction of total EGFR-3M (L858R/T790M/C797S) proteins through STUB1-mediated ubiquitination and degradation	^173^
		Castration resistant prostate cancer	Docetaxel	Suppression of the expression and nuclear translocation of AR-FL and AR-Vs	^174^
		Triple-negative breast cancer	Anti-PD-L1 Ab	Increase in sensitivity to anti-PD-L1 therapy by increasing PD-L1 expression and downregulation of CD4^+^ FOXP3^+^ Treg	^156^
	Belinostat	NSCLS (cisplatin-resistant)	Cisplatin	Increase in the association of a transcriptional repressor to the *ABCC2* gene	^175^
		Advanced HCC	Anti-CTLA4 ab	Enhanced IFN-γ production by antitumor T cells and a decrease in regulatory T cells	^176^
		Acute promyelocytic leukemia	All-trans-retinoic acid	Downregulation of EZH2, SUZ12, HDAC-1, HDAC-2, and PCAF expression	^177^
	Panobinostat	DLBCL	Ibrutinib	Decreased phosphorylated STAT3 binding to the *MyD88* promotor, downregulation of MyD88 expression, and inhibition of NF-κB activity	^178^
		Acute myeloid leukemia (adriamycin-resistant)	Bortezomib	Induction of apoptosis by inhibiting the PI3K/AKT and NF-κB pathway	^179^
		Triple-negative breast cancer	NA	Upregulation of CDH1 protein expression and reversal of the mesenchymal phenotype	^180^
		Neuroblastoma (MYCN-amplified)	JQ1	Downregulation of LIN28B gene and N-MYC protein expression	^181^
		Breast cancer (trastuzumab-refractory)	Trastuzumab	Abrogation of AKT signaling and induction of CXCR3-reactive ligand expression	^182^
		NSCLC (KRAS-mutated)	Gefitinib	Downregulation of TAZ downstream targets, including EGFR and EGFR ligand	^183^
		Castration resistant prostate cancer	Bicalutamide	Reduction of AR-mediated resistance to bicalutamide	^184^
		EGFR-Mutant NSCLC (osimertinib-resistant)	Osimertinib	Elevation of BIM in osimertinib-resistant cells	^185^
		Melanoma	Anti-PD-(L)1 Ab	Increase in sensitivity to anti-PD-L1 therapy by induction of PD-L1 and PD-L2 expression	^154^
Class I HDACs	Entinostat	Bladder, colorectal, and breast cancers	Anti-PD-1 Ab	Promotion of immune editing of tumor neoantigens, effectively remodeling the tumor immune microenvironment	^159,161^
	Romidepsin	Peripheral T-cell lymphoma	Tamoxifen	Induction of apoptosis by activating FOXO1 expression	^186^
		Lung cancer	Anti-PD-1 Ab	Upregulation of chemokine (CXCL9, CXCL10), PD-1L, and STAT1 expression.	^152^
		Triple-negative breast cancer Clear cell renal cell carcinoma	Decitabine	Re-expression of the tumor suppressor gene (sFRP1)	^187^
Class I/II HDACs	Givinostat (ITF-2358)	NSCLC	Aazacitidine (DNMTi)	Depletion of MYC in NSCLC and enhancement of NSCLC sensitivity to HDACi, Upregulation of T-cell chemoattractant (CCL5) expression	^158^
HDAC6	Nexturastat A	Melanoma	Anti-PD-1 Ab	Complete neutralization of the upregulation of PD-L1 and other immunosuppressive pathways induced by the treatment with anti-PD-1 blockade	^162^
	Citarinostat	Multiple myeloma	NA	Upregulation of B7 (CD80, CD86) and MHC-I,II expression in tumor and dendritic cells	^163^
HDAC11	FT234	NSCLC (erlotinib-resistant)	NA	Suppression of GLI1-mediated SOX2 expression	^120^
SIRT1	Tenovin-6	Chronic myeloid leukemia	Imatinib	Promotion of p53 acetylation and transcriptional activity	^123^

*NSCLC* non-small-cell lung cancer, *HCC* hepatocellular carcinoma, *DLBCL* diffuse large B-cell lymphoma, *NA* not applicable.

## Histone modifications in immune evasion of CSC

### Histone methylation

Several theories have been proposed to explain the unresponsiveness or evasion of ICIs, including intrinsic tumor modifications such as the lack of neoantigens, inactivation of the antigen presentation machinery, blocking of IFN-γ signaling, and extrinsic tumor modifications such as the loss of antigen-presenting cell function through the release of immunosuppressive cytokines, decreased T-cell proliferation and cytokine release, upregulation of additional ICs (e.g., TIM3 and LAG3), and activation of Tregs and MDSCs^126,127^. Histone methylation is directly involved in immune evasion mechanisms of cancer cells, including breast, prostate, colorectal cancers, and AML^6,7^, leading to new strategies to regulate the balance of histone methylation for cancer immunotherapy.

G9a and SETDB1 play essential roles in the regulation of pluripotency, stemness, and tumorigenicity^128,129^. Notably, the expression levels of G9a and SETDB1 correlated with the response to anti-PD-1 immunotherapy. For example, in a mouse bladder cancer model, the dual inhibition of G9a and DNA methyltransferase (DNMT) enhanced the response to anti-PD-L1 therapy, increased the tumor infiltration of NK cells and CD8^+^ T cells, and facilitated tumor regression^130^. In addition, inhibition of G9a in neuroblastoma can enhance IFN-γ-stimulated expression of CXCL9 and CXCL10, which are important Th1 chemokines for the recruitment of cytotoxic T cells to the TME of neuroblastoma^131^. The dual inhibition of histone modification enzymes has also been evaluated. In multiple myeloma cells, simultaneous inhibition of G9a and EZH2 induces the expression of ERV factors and activates the IFN response, causing cell cycle arrest and apoptosis^132^. In cancer, overexpression of SETDB1 negatively correlates with a characteristic gene signature associated with a positive immune response. Patients with renal cell carcinoma with poor survival response to anti-PD-1 therapy have been shown to have amplification of the *SETDB1* gene^133^. Consistently, *SETDB1* knockout in a melanoma mouse model induced the re-expression of ERV antigens presented on the surface of cells by MHC-I to induce a cytotoxic T-cell response. Another study showed that JARID1B (KDM5B), an H3K4 demethylase, can assist SETDB1 by recruiting SETDB1 to the endogenous retrovirus (ERV) element site^134^, suggesting that SETDB1 is a potential target to restore the immune surveillance of CSCs.

EZH2 has received increased attention as a cause of resistance in tumor immunotherapy because it is overexpressed in various cancer types, including melanoma, and silences tumor-suppressor genes or genes associated with antigen presentation^135^. The increased expression of EZH2 is associated with the promotion of Treg cell differentiation^136^, while it is inversely correlated with tumor infiltration of CD8^+^ T cells^137^. In a melanoma mouse model, EZH2 was associated with the epigenetic development of resistance against recombinant IL-2 (rIL2) and ICI. Inhibition of EZH2 activity concurrently with rIL2/anti-CTLA-4 immunotherapy downregulates the expression of PD-L1, promotes the expression of IFN-γ, increases the number of intratumoral PD-1^low^TIM-3^low^LAG-3^low^CD8^+^ T cells, and reverses resistance against anti-CTLA-4 and IL-2 immunotherapies^135^. EZH2 inhibitors also restore the expression of Th1-type chemokines in ovarian cancer, facilitate tumor infiltration of effector T cells, and enhance the efficacy of anti-PD-L1 treatment^137^. In contrast to melanoma, which shows high PD-L1 expression, low PD-L1 expression in cancer cells is highly associated with low responsiveness to anti-PD-(L)1 therapy. Xiao et al. showed that EZH2 in hepatocellular carcinoma directly upregulated the level of H3K27me3 at the *CD274* promoter to reduce PD-L1 expression^138^. In addition, EZH2 contributes to the progression of hepatocellular carcinoma by increasing the expression of additional immune checkpoints such as Gal-9 and TIM-3 ligand through H3K27 trimethylation at the *miR-22* promoter, with a positive role in the progression of hepatocellular carcinoma^139^. Goswami et al. observed that peripheral T cells in patients treated with anti-CTLA-4 therapy (ipilimumab) had increased EZH2 expression^140^. In the same study, the administration of an EZH2 inhibitor (CPI-1205) in bladder cancer and melanoma mouse models increased the cytotoxic activity of CD8^+^ effector T cells, altered the phenotype and function of Tregs, and increased susceptibility to ipilimumab, providing a rationale for combining immune checkpoint inhibitors and histone modification inhibitors against ICI-resistant cancers (Table 1).

In pancreatic ductal adenocarcinoma, attempts to inhibit PRMT1 to overcome drug resistance against anti-PD-L1 therapy led to a positive outcome^141^. Unexpectedly, the inhibition of PRMT5 in lung cancer increases *CD274* gene expression, eventually activating the PD1/PD-L1 axis and blocking T-cell-mediated antitumor immunity^142^. Thus, PRMT5 inhibition in combination with anti-PD-L1 therapy could be a breakthrough even though further research is required to investigate its underlying activities.

Elevated LSD1 expression in breast, lung, and other cancer types is not only essential in maintaining CSC stemness and mediating chemoresistance^143^ but is also inversely proportional to the expression levels of T-cell-attracting chemokines (e.g., CXCL9 and CXCL10) and CD8^+^ T-cell infiltration, which predicts a poor prognosis in clinical practice^144,145^. In stem-like cells of sorafenib-resistant hepatocellular carcinoma, LDS1 inhibitors block WNT/β-catenin signaling to overcome drug resistance^146^. In addition, LSD1 suppresses IFN-mediated antitumor immunity by preventing the activation of ERV factors through demethylation of Argonaute RISC catalytic component 2 (AGO2)^144^. Hence, LSD1 inhibitors exhibit antitumor effects by derepressing ERV factors, activating type I IFN expression, activating CD8^+^ T cells, facilitating infiltration in cancer tissues, and promoting DC differentiation in melanoma^147^. The effect of LSD1 inhibition in overcoming unresponsiveness to ICIs has been verified in various models. In melanoma and TNBC mouse xenograft models, LSD1 inhibitors in combination with anti-PD-1 antibodies increase CD8^+^ T-cell infiltration and significantly suppress tumor growth and lung metastasis^147^. The inhibition of LSD1 also enhanced the efficacy of ICI treatment in HNSCC and cervical cancer mouse models^148,149^. Of note, however, is that the removal of LSD1 in melanoma induced the expression of TGF-β, which suppresses T-cell immunity; as a result, the tumor could not be completely eradicated despite the enhanced efficacy of the combination with ICIs^144,147^. As a solution, Sheng et al.^147^ suggested that in ICI treatments, simultaneous inhibition of LSD1 and TGF-β could contribute to the complete eradication of ICI-refractory tumors and prevent tumor recurrence. These findings suggest that the abovementioned triple combination could be applied to the treatment of other types of tumors with low immunogenicity.

### Histone acetylation

In various tumor mouse models, treatment with HDAC inhibitors has shown interesting effects in reversing drug resistance against ICIs through varying mechanisms^150^. Inhibition of HDAC using romidepsin (a class I HDACi) in a melanoma mouse model increased the expression of genes related to MHC I antigen processing and presentation, including transporter 1, ATP binding cassette subfamily B member (TAP1), proteasome 20S subunit beta 9 (LMP2), and β-2-microglobulin (B2M), and improved the cytotoxic activity of CD8^+^ T cells^151^. The use of romidepsin in a lung tumor model increased the response to PD-1 blockade immunotherapy and improved T-cell infiltration^152^. Additionally, Woods et al. showed that panobinostat (pan-HDACi) in melanoma increased the expression of MHC-I in cancer cells and increased the production of IL-2 and IFN-γ from CD4^+^ T cells, subsequently increasing the sensitivity to anti-PD-(L)1 therapy^153,154^. Panobinostat regulates the levels of various cytokines associated with T-cell activation in patients with Hodgkin’s lymphoma^155^. Vorinostat (a pan-HDACi) increases sensitivity to anti-PD-L1 therapy by increasing PD-L1 expression and downregulating CD4^+^ FOXP3^+^ Tregs in TNBC^156^. Similarly, Briere et al. reported that in a syngeneic tumor model, a class I/IV HDACi (mocetinostat) increased PD-L1 expression and exhibited a significantly improved antitumor immune response in combination with anti-PD-L1 therapy^157^. In addition, givinostat, a class I/II HDAC inhibitor, blocked tumor regression and metastasis when used with DNMTi (azacitidine), anti-CTLA-4 mAb, or anti-PD-1 mAb in an NSCLC model^158^. Entinostat (a class I HDACi) inhibited the function of MDSCs and increased the antitumor effect of anti-PD-1 therapy in metastatic BC^159^. In other studies, entinostat inhibited MDSCs in lung cancer and renal cell carcinoma mouse models to reinforce the effects of anti-PD1 therapy^160^ and induce the expression of genes related to antigen presentation in BC^161^. In addition, Knox et al. reported that treatment with a selective HDAC6i (nexturastat A) increased IFN-γ and IL-2 expression in the SM1 murine melanoma model and improved the TME to maximize the effects of anti-PD-1 therapy^162^. ACY241, another HDAC6 inhibitor, upregulated the expression of costimulatory (CD28 and CD40L) and activating (CD38, CD69, and CD137) molecules in bone marrow cells in multiple myeloma and increased T-cell activity^163^. To date, clinical studies on the combined use of HDAC inhibitors and immune checkpoint inhibitors have been positive. However, some HDAC inhibitors also have negative effects on immune cell viability and function that possibly limit the effect of immune therapy, such as inhibiting the activation and proliferation of lymphocytes, thereby requiring further investigation^164,165^.

Unlike HDAC inhibitors, the positive effects of HAT inhibitors have not yet been reported. Nevertheless, HAT also has the potential to become an important target molecule for immunotherapy. Fan et al. reported that an upregulated level of HAT1 (histone acetyltransferase 1, KAT1) was correlated with a poor prognosis of pancreatic cancer^166^. The study also showed that HAT1 regulated the expression of PD-L1 at the transcriptional level in both in vitro and in vivo models and that HAT1 knockdown reduced pancreatic tumor cell proliferation. In addition, PD-L1 expression was positively correlated with HAT1 expression in pancreatic tumor tissues. These results indicate that in patients with acquired resistance due to abnormal expression of HAT1 and PD-L1, targeting HAT1 activity could maximize the antitumor immune response to ICIs by blocking the PD-1/PD-L1 axis and increasing sensitivity to ICIs.

**Fig. 3:**
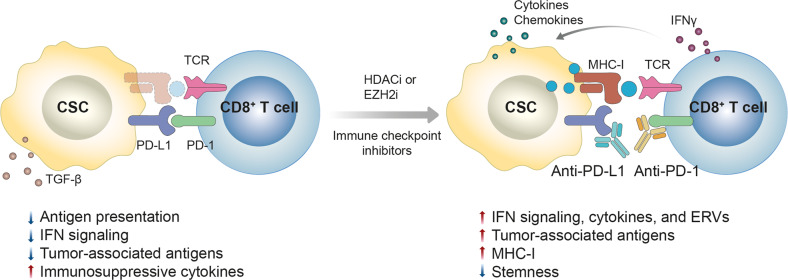
Overcoming cancer resistance to immune checkpoint inhibitors using histone modification inhibitors. The expression of PD-L1 and tumor mutational burden (TMB) in tumors are critical factors for responsiveness to immunotherapy. TMB increases the probability of neo-antigen expression and eventually leads to an effective immune response. Histone-modifying enzymes (e.g., HDACs and EZH2), which are overexpressed in CSCs, enhance stemness and decrease immunogenicity through epigenetic modulation in CSCs, making them resistant to immunotherapy. This tumor immune evasion is acquired through the decreased expression of neoantigens, secretion of immunosuppressive cytokines, decreased antigen processing and presentation, and attenuation of IFN signaling. Inhibitors of histone-modifying enzymes promote the expression of neoantigens, activate the antigen presentation pathway, increase the secretion of immune-enhancing cytokines (e.g., IFNs) in resistant tumors, and downregulate immunosuppressive factors in the TME, leading to tumor rerecognition by the immune system and increasing the efficacy of anti-PD-(L)1 therapy.

These results collectively suggest that histone modification enzymes regulate the adaptive resistance mechanisms against tumor immunotherapy to act as epigenetic checkpoints to suppress immune responses and that these histone modifiers could serve as potential therapeutic targets for the improvement of immunotherapy (Fig. 3).

## Inhibitors for histone modificatons

As previously mentioned, epigenetic mechanisms exert a significant impact on both host immune cells and tumor cells, and epigenetic drugs enhance the efficacy of cancer immunotherapy through various mechanisms. In line with this, the combination of conventional anticancer chemotherapy drugs or ICIs with histone modification inhibitors is highly attractive, with dramatic activation effects, as shown in numerous studies using cellular and animal models. Combined treatments with histone modification inhibitors and other anticancer drugs are currently being tested in studies targeting various cancer types.

The efforts of the past two decades have been successful, and several histone deacetylation inhibitors (HDACis) and histone methylation inhibitors (EZH2is) have been clinically approved. Vorinostat was the first HDAC inhibitor approved by the FDA in 2006 for the treatment of cutaneous T-cell lymphoma (CTCL). Romidepsin is an HDAC inhibitor approved in 2009 for the treatment of CTCL and peripheral T-cell lymphoma. Belinostat is the third HDAC inhibitor approved for the treatment of peripheral T-cell lymphoma. Subsequently, panobinostat was approved for the treatment of multiple myeloma in 2015. In 2020, tazemetostat, an EZH2 inhibitor, was approved for the treatment of epithelioid sarcoma, making it the first approved histone “writer” inhibitor and the first used to treat solid tumors^167^. Most recently, the EZH1/2 dual inhibitor valemetostat was approved for the first time in Japan for the treatment of adult T-cell leukemia/lymphoma, for which there is no suitable therapeutic option^168^. The recent results of clinical trials of histone modification inhibitors alone or in combination with chemotherapy or immunotherapy for the treatment of drug-resistant cancer are summarized in Table 2.

**Table 2:** Recent results of clinical trials using histone modification inhibitors alone or in combination with chemo- or immunotherapy for the treatment of drug-resistant cancers.

Inhibitor	Target	Combination therapy	Cancer type	Phase	Endpoint outcome	Clinical trial ID
Vorinostat	HDACs	Hydroxychloroquine, Regorafenib	Chemotherapy-refractory metastatic colorectal cancer	II	Not achieved	NCT02316340
		NA	Recurrent/metastatic transitional cell carcinoma of the urothelium	II	Not achieved	NCT00363883
		Bortezomib, dexamethasone	Relapsed myeloma	II	Achieved	ISRCTN08577602
		Pembrolizumab	Recurrent/metastatic HNSCC and salivary gland cancer	II	Achieved	NCT02538510.
		Bevacizumab	Recurrent glioblastoma	II	Not achieved	NCT01266031
		Pembrolizumab	Advanced/metastatic non-small-cell lung cancer	I/IIb	Achieved	NCT02638090
		Bortezomib	Mantle Cell Lymphoma	II	Achieved	NCT00703664
		Rituximab-CHOP	Advanced diffuse large B-cell lymphoma	I/II	Not achieved	NCT00972478
		Bevacizumab	Grade 4 malignant glioma	II	Not achieved	NCT01738646
Romidepsin	Class I HDACs	5-Azacytidine	Peripheral T-cell lymphoma	II	Achieved	NCT01998035
		Lenalidomide	Relapsed/refractory lymphoma	I	Achieved	NCT01755975, 02341014
		Alisertib	Relapsed/refractory aggressive B-cell and T-cell lymphoma	I	Achieved	NCT01897012
Belinostat	HDACs	Followed by zevalin	Relapsed aggressive high-risk lymphoma	II	Not achieved	NCT01686165
		Bortezomib	Released/refractory acute leukemia, myelodysplastic syndrome	I	Not achieved	NCT01075425
		Cisplatin and etoposide	Small-cell lung cancer	I	Achieved	NCT00926640
Panobinostat	HDACs	Everolimus	Relapsed/Refractory Diffuse Large B-Cell Lymphoma	II	Not achieved	NCT00978432
		Bortezomib and dexamethasone	Relapsed/Refractory Multiple Myeloma	II	Achieved	NCT02290431
		NA	Graft-versus-host disease	II	Achieved	NCT02588339
		NA	Multiple myeloma (a complete response failed after transplantation)	II	Achieved	ACTRN12613000219785
		Bortezomib and dexamethasone	Relapsed/refractory multiple myeloma	II	Achieved	NCT02654990
		Carfilzomib	Relapsed/refractory multiple myeloma	I/II	Achieved	NCT01496118
Tazemetostat	EZH2	NA	Relapsed/refractory, BAP1-inactivated malignant pleural mesothelioma	II	Not achieved	NCT02860286
		Atezolizumab	Relapsed/refractory diffuse large B-cell lymphoma	Ib	Achieved	NCT02220842
		NA	Relapsed/refractory B-cell non-Hodgkin lymphoma with EZH2 mutation	II	Achieved	NCT03456726
		NA	Relapsed/refractory follicular lymphoma	II	Achieved	NCT01897571

*CHOP* cyclophosphamide, doxorubicin, vincristine, and prednisone, *HNSCC* head and neck squamous cell carcinoma, *NA* not applicable.

## Conclusion

Epigenetic alterations influence normal gene expression and consequently play a critical role in the onset and progression of diverse cancer types. CSCs are the main cause of cancer recurrence, metastasis, and treatment failure. The mechanisms that enable the characteristic drug resistance and immune evasion of CSCs are influenced by epigenetic regulation. These epigenetic modifications can be altered or reversed, thus ensuring new promising strategies in the therapeutic approach to CSCs. While advancements over the past several decades have yielded information to overcome drug-resistant cancer through the control of histone modification, several questions remain unanswered. Histone-modifying enzymes extensively remodel the TME to enable cancer cells to resist various anticancer therapies. Thus, the role of histone modification inhibitors should include not only blocking the intrinsic function of CSCs, which attenuates the antitumor response but also interfering with CSC crosstalk with immune cells to restore immune cell activity, ultimately converting aggressive CSCs into controllable “naïve” cells through complete reorganization of the epigenetic landscape. Despite the positive results of the combination of histone-modifying enzyme inhibitors for synergistic activity of tumor immune therapies, the diversity of genes targeted by these inhibitors makes it difficult to fully elucidate the mechanisms underlying complementary or synergistic actions with ICIs in the TME. In addition, approaches to cancer types in which the effects of histone modification inhibitors are relatively poor must be addressed. Because the combination effect with other anticancer drugs is maximized through the formation of suitable CSCs or immunological conditions via epigenetic priming by histone modification inhibitors, suitable biomarkers need to be identified to achieve a satisfactory combination effect. Nonetheless, modulation of histone modifications involved in CSC drug resistance and immune evasion will maximize clinical benefits through appropriate chemotherapeutic and immunotherapeutic combinatorial approaches, paving the way for personalized precision medicine.
